# Editorial: Immune intervention avenues to control fungal infections in immunocompromised individuals

**DOI:** 10.3389/fimmu.2023.1212797

**Published:** 2023-09-18

**Authors:** Som G. Nanjappa

**Affiliations:** Department of Pathobiology, College of Veterinary Medicine, University of Illinois at Urbana-Champaign, Urbana, IL, United States

**Keywords:** immunocompromised, fungal, immunity, interventions, COVID-19, vaccine, therapeutics

Immune intervention avenues to control fungal infections in immunocompromised individuals

Among millions of fungal species, only a few account for most infections in humans. The occurrence primarily depends on the host’s immune status, and deficiency or suppression or compromised immune system enhances the susceptibility and disease severity in the affected individuals. The most devastating feature of fungal diseases, distinct from many other pathogens, is their high case fatality rate, ranging from 25% to 95% (1). Recent pandemic COVID-19-associated severe fungal infections shocked the scientific and healthcare community (2). In this Research Topic, four articles describe COVID-19-associated fungal comorbidities and potential immune- preventive and therapeutic targets/strategies.

The ever-accumulating research knowledge on immunity to fungal infections is remarkable (3). Although few fungal species are pathogenic, the protective immune elements or their products are diverse and often complementary, mediated by CD4^+^ T, CD8^+^ T, and B cells (4, 5). Immunity to histoplasmosis, cryptococcosis, paracoccidioidosis, and aspergillosis is primarily by type I (IFNγ, GM-CSF, TNF) T cell responses. In contrast, immunity to blastomycosis, coccidioidomycosis, and mucosal candidiasis, predominantly depends on type 17 responses and type 2 responses are largely considered non-protective. Although a pan-fungal vaccine is ideal, the distinct protective responses to different pathogenic fungi need attention. Nevertheless, fungal vaccines, including cross-reactive targeting of multiple species within the genera, are beneficial to ameliorate or prevent disease and the emergence of drug resistance (Hester et al.). The study by Hester et al. showed that experimental vaccination with a single chitin deacetylase (Cda) protein can cross-protect against the multiple strains of *Cryptococcus neoformans* including those lacking the vaccine Cda. The immunity was mediated by cross-reactive antibodies and IFNγ responses to Cda member proteins, and loss of CD4^+^ T cells abrogated the vaccine immunity. Although CD8^+^ T cells are implicated in immunity to fungal infections (Sharma et al.), the vaccine Cda does not seem to induce threshold immunity provided by them.

The recent COVID-19 pandemic outbreak revealed the wrath of fungal infections, with case fatality rates surpassing 80% (2). Although the immunosuppressive corticosteroids used to mitigate hyperinflammation and underlying risk factors including diabetes precipitate fungal infections in COVID-19 patients, several other factors may influence their occurrence or severity (Tappe et al.). Here, Tappe et al. showed that innate and T cell-derived inflammatory cytokines were diminished in COVID-19 patients with *Aspergillus fumigatus* and *Rhizopus arrhizus* infections. COVID-19 patients with fungal infection displayed higher levels of the T-cell exhaustion marker PD-1 and weakened chemokine and cytokine responses to fungal antigens by granulocytes/dendritic cells. Based on these observations, this study proposed a model outlining the putative mechanisms of increased susceptibility to fungal pathogens in COVID-19 patients. In line with this (Morton et al.), Morton et al. reviewed the literature and identified various risk factors for invasive fungal diseases in COVID-19 patients. These risk factors included: the virus-induced elements, including loss of barrier integrity, dysregulated T cell responses, reduced numbers or functions of NK cells, T cells, dendritic cells, neutrophils; therapeutics induced elements such as immune suppressive treatments; and genetic polymorphisms that led to enhanced susceptibility to fungal infections. While fungal infections are primarily controlled by chemotherapeutics, which are highly toxic and prone to emergence of resistance, immunotherapeutics are attractive and necessary alternatives (Wurster et al.). Wurster et al. showed the upregulation of various co-inhibitory molecules (immune checkpoints) of T cells during different fungal infections. The review pointed out various potential avenues of immune interventions to bolster antifungal immune responses. Additionally, it provided a few clinical examples where such interventions might help patients recover from fungal diseases.

This Research Topic collated articles on corticosteroid-independent and generalized impaired immunity to pathogenic fungi in COVID-19 patients, checkpoint inhibitors as immunotherapeutic agents for fungal infections, and a potential cross-protective subunit cryptococcus vaccine. Novel immune intervention strategies are necessary to control fungal infections in immune-compromised individuals. Subunit-safe vaccines encompassing immunogenicity to protect against broad species are highly desirable preventive measures. As therapeutic measures, immune restoration of exhausted antifungal T cells or suppressed innate immune cells by countering the inhibitory signals help bolster antifungal immunity in COVID-19 patients. Given the increasing immunocompromised population in recent years, these novel avenues offer long-lasting thrust to combat deadly fungal infections.

## Author contributions

The author confirms being the sole contributor of this work and has approved it for publication.
